# Model Based Analysis of Clonal Developments Allows for Early Detection of Monoclonal Conversion and Leukemia

**DOI:** 10.1371/journal.pone.0165129

**Published:** 2016-10-20

**Authors:** Christoph Baldow, Lars Thielecke, Ingmar Glauche

**Affiliations:** Institute for Medical Informatics and Biometry, Carl Gustav Carus Faculty of Medicine, Technische Universität Dresden, Dresden, Germany; B.C. Cancer Agency, CANADA

## Abstract

The availability of several methods to unambiguously mark individual cells has strongly fostered the understanding of clonal developments in hematopoiesis and other stem cell driven regenerative tissues. While cellular barcoding is the method of choice for experimental studies, patients that underwent gene therapy carry a unique insertional mark within the transplanted cells originating from the integration of the retroviral vector. Close monitoring of such patients allows accessing their clonal dynamics, however, the early detection of events that predict monoclonal conversion and potentially the onset of leukemia are beneficial for treatment. We developed a simple mathematical model of a self-stabilizing hematopoietic stem cell population to generate a wide range of possible clonal developments, reproducing typical, experimentally and clinically observed scenarios. We use the resulting model scenarios to suggest and test a set of statistical measures that should allow for an interpretation and classification of relevant clonal dynamics. Apart from the assessment of several established diversity indices we suggest a measure that quantifies the extension to which the increase in the size of one clone is attributed to the total loss in the size of all other clones. By evaluating the change in relative clone sizes between consecutive measurements, the suggested measure, referred to as *maximum relative clonal expansion* (*mRCE*), proves to be highly sensitive in the detection of rapidly expanding cell clones prior to their dominant manifestation. This predictive potential places the *mRCE* as a suitable means for the early recognition of leukemogenesis especially in gene therapy patients that are closely monitored. Our model based approach illustrates how simulation studies can actively support the design and evaluation of preclinical strategies for the analysis and risk evaluation of clonal developments.

## Introduction

The life-long supply of functional blood cells is realized by a rather small population of hematopoietic stem cells (HSCs). These cells reside in the bone marrow and proliferate with a relatively low frequency [1–3]. It was shown in different experiments that individual HSCs are able to fully regenerate adult hematopoiesis after stem cell depletion, thus documenting that individual HSCs can expand and reestablish their own population [4, 5]. In addition to this self-renewal potential the HSC pool in human generates a vast amount of functional blood cells every day in a tightly regulated sequence of amplification steps [6, 7].

In a simple approximation, the descendants of each HSC constitute a particular clone, which can both expand within the HSC pool and contribute to the pool of more differentiated progeny and therefore to the peripheral blood [8]. Although the terminology *clone* is commonly used in stem cell research we advocate the view that it should be handled with care and provided with defining criteria. Originally the term derives from the Greek word κλω ´ν (twig) and refers to the process of deriving new plants by implanting twigs. In this meaning of the word, it is the common ancestor that uniquely defines a clone by the set of its descendants. In cell biology this definition has been adapted to the cellular offspring of an ancestral cell [9]. Whether this initial cell is defined a priori (e.g. as a fertilized egg), by a marking event (e.g. integration of a unique vector) or simply by the initiation of recording (e.g. in single cell tracking) depends on the particular experimental view and the raised question.

HSCs contribute to the blood in cases of demand and otherwise only infrequently [10]. In any case, the development of one individual clone can never be seen as an independent process as it is ultimately linked to the development and the expansion of other, possibly competing clones. This process is usually referred to as clonal competition or clonal dynamics [9, 11].

Clonal competition occurs due to HSC clones that have slightly different properties while competing for similar *resources*, often interpreted as a competition for niche spaces [12]. Assuming an intrinsic heterogeneity of all HSCs, clonal competition appears as a continuous process that defines the clonal repertoire in the long run [13]. Blood cancers, like myeloid leukemia, disturb this natural, mild heterogeneity by the (possibly sequential) generation of clones with a distinct growth advantage and impaired differentiation potential [14–17]. This leads to an increased and unregulated expansion of predominantly immature myeloid cells, the out-competition of healthy cells and finally, if untreated, to the patient’s death. The question arises, whether it is possible to identify the dominant growth of a cancer clone already at an early stage in which the pathological potential is not fully developed and additional mutation acquisition is still limited. Since clonal fluctuations are observable even in healthy tissues the identification of a maligned/leukemic clone based on fluctuation patterns remains challenging.

The analysis of clonal developments in hematopoiesis has evolved into a key method in both experimental and clinical research. Established methods are based on cell intrinsic information, which can be used to conclude divisional history of the corresponding cells, e.g. gene mutation profiles, without alternating the cell during the experiment or therapy [11]. The availability of sophisticated methods to uniquely label individual HSCs and their clonal progeny offers an exciting experimental access to such data. Mainly the use of barcode equipped viral vectors in combination with next generation sequencing significantly foster the experimental progress. Although the use of such viral vectors in clinical studies is limited by ethical and safety constrains, clonal tracking using integration site analysis is the method of choice for the close monitoring of gene therapy patients [18–20]. Particularly in this settings, it is of highest relevance to utilize the temporal clonal development in individual patients to potentially identify malignant transformations at a very early point in time and to reliably and prospectively predict the potential outgrowth of leukemia [19]. This would allow for the early adaptation of treatment strategies; such as stem cell transplantations.

We developed a simplified, albeit rigorously defined mathematical model to study how changes in the clonal properties influence the dynamics of clonal evolution. We use the model to simulate clonal behavior in the hematopoietic stem cell compartments in order to illustrate how certain aspects of the experimental setup (such as number and size of initially transplanted cell, inter-cellular variability, sampling and measurement errors) influence the resulting clonal patterns of hematopoiesis. Furthermore, we study how the rapid outgrowth of a leukemic clone influences the clonal competition process.

It is the central aim of our study to systematically analyze how a range of statistical measures is suited to quantify clone size distribution and abundance over time. We are particularly interested in the performance evaluation of such measures especially for the case of a leukemic clone and whether they can be used to reliably and prospectively detect the occurrence of such malignancies. We discuss potential applications in the context of gene therapies.

## Material and Methods

### Simplified Model of Hematopoiesis

We apply an agent-based model, in which cells are represented by individual agents and are updated at discrete time steps (daily). Every cell has several characteristic attributes (clonal identity *i*, proliferation rate *p*_*i*_, differentiation rate *d*_*i*_, replicative age $\delta_{c_{i}}$; see below) that are inherited to their progeny.

The population growth is limited by a logistic growth function with carrying capacity *K*. Proliferation thereby only depends on the absolute cell number and is modeled as a stochastic update procedure, in which the probability of a proliferation event $\Omega_{p}^{c}$ for a single cell *c* at time t is given as

$$P\left(\Omega_{p}^{c},\;t\right)=p_{i}\cdot \left(1-\frac{N\left(t\right)}{K}\right)$$

Herein *p*_*i*_ is the maximum proliferation rate for the scenario of an empty model system. For simplicity we assume that the proliferation rate is identical for all healthy clones (*p*_*i*_ = *p*) whereas this value is increased for malignant clones (see blow). Due to the implementation of proliferation as a stochastic process the inverse of the average probability $\left(1/\bar{P\left(\Omega_{p}^{c},t\right)}\right)$ corresponds to an effective cell turnover time. {*N*_1_,*N*_2_, …, *N*_*i*_, …*N*_*n*_} defines a set of *n* different clones, in which the cells share and inherit common clonal properties (namely differentiation and proliferation rates). Every cell *c* is assigned to a certain clone *i*, which is inherited to their clonal offspring. $N\left(t\right)=\sum_{i=1}^{n}\left|N_{i}^{t}\right|$ refers to the total number of cells at time point t within all clones.

Besides proliferation, a cell *c* can also undergo differentiation $\Omega_{d}^{c}$ at time point *t* with the probability

$$P\left(\Omega_{d}^{c},\;t\right)=d_{i}\cdot \left(1+\alpha \cdot \delta_{c}\left(t\right)\right)$$

with $d_{i}\;\sim \;N\left(d,\;\sigma^{2}\right)$ being a clone specific differentiation rate of clone *i*. Initially, values of *d*_*i*_ are drawn from a normal distribution with mean *d* and standard deviation *σ*, thereby mimicking a degree of clonal heterogeneity.

We represent aging as the cumulative adverse effect resulting from multiple replications. Technically, the replicative age *δ*_*c*_ counts the sum of all prior divisions of a certain cell *c*, and therefore increasing the differentiation probability by a factor termed *aging factor α*. For the limiting case *α* = 0 the aging effect is neglected, and therefore only the differentiation rate *d*_*i*_ makes up for the clonal differences.

Furthermore, we incorporate cancer in the model as a “one hit model”, in which we make the simplifying assumption that a single mutation event alters the proliferation rate of one randomly chosen cell. Technically, a new clone *N*_*n*+1_ is derived from this randomly chosen cell, thereby constituting an independent subclone of the initially marked clone to which the affected cell belongs. All offspring cells of the new clone *N*_*n*+1_ are characterized by an increased proliferation rate *p*_*n*+1_ ≫ *p*_*i*_ with *i* ∈ {1, …, *n*}. Since we are using a logistic growth limitation to describe the population maintenance, the mutated cell clone can eventually outcompete the non-mutated, “healthy” clones.

Uncertainty of the measurement process is incorporated within our model by adding a white noise N(μ=0,η) to the absolute clone size measurements. The standard deviation *η* refers to the noise amplitude. The case *η* = 0 corresponds to a scenario without any measurement noise.

Clone size analysis is performed directly within the population of proliferative and differentiating (stem) cells, thereby neglecting alterations due to differential amplification of certain cell clones. However, without any further assumptions, down-stream compartments of the stem cell pool will only reflect the clonal composition with a moderate time delay.

The simulation framework is implemented as a single-cell based model in R (version 3.3.1). Further details of the simulation sequence are outlined in S1 Code.

### Choice of parameters

We have adapted our model to resemble plausible homeostatic and leukemic developments within appropriate mouse models. In the following, we use the outlined standard configuration:

- We set the carrying capacity to *K* = 2000 stem cells, thereby estimating the size of a mouse HSC compartment [21]. Upscaling to higher cell numbers does not qualitatively change the presented results.
- We report our results for the case of *n* = 20 clones. This value was chosen for illustrative reasons, although we are aware that we are underestimating the true number of marked HSC clones within a mouse. Again, upscaling to higher clone numbers does not qualitatively alter our results.
- We assume that cell division occurs randomly. The effective proliferation rate is calculated as the product of an maximum proliferation rate of (10 *days*)^−1^ for healthy cells and (6.6 *days*)^−1^ for malignant cells and a logistic growth limitation. The maximum proliferation rate corresponds to earlier estimates for the turnover of activated HSCs [1, 3]. Compared to a small fraction of deeply quiescent (label-retaining) HSCs this is a rather high frequency, however, we argue that precisely this population of activated cells contributes to peripheral blood while still retaining self-maintenance potential [1, 2].
- Cell differentiation (i.e. loss of stem cell potential) occurs randomly with a clone specific rate $d_{i}\;\sim \;N\left(d,\;\sigma^{2}\right)$. The value for the expected differentiation rate is chosen as *d* = 0.03 representing a typical outflux of short term HSCs [2].
- In order to consider an additional aging influence, we increase the differentiation probability with every division of a cell’s predecessors. We tuned the magnitude of the aging effect to *α* = 0.01 such that oligoclonality occurs after about ten years (compare earlier estimates [22]).

### Measures

Clonal contribution is characterized by the number of contributing clones and their abundancy within certain cell compartments. In order to quantify and characterize the temporal change of these contributions, we apply a range of diversity measures that have been used successfully in various research fields to describe population inherent heterogeneities.

- *Species richness* (SR) describes the diversity of species in a defined environment. In particular, it counts the number of different clones and does not take their abundances into account. Here, we use a normalized version of this measure, in which the maximum number of different clones *n* is considered [23]:
  $$SR\left(t\right)=1-\frac{\sum_{i=1,\;\left|N_{i}^{t}\right|>0}^{n}1}{n}$$
- *Simpson index* (SI) describes the probability that two randomly chosen individuals do not belong to the same species within the population. In our application this translates to the probability that two cells from the population do not belong to the same clone. If this index reaches 1 there is no diversity, meaning the population consists of just one clone [24, 25].
  $$SI\left(t\right)=1-\;\sum_{i=1}^{n}\frac{\left|N_{i}^{t}\right|\left(\left|N_{i}^{t}\right|-1\right)}{N\left(t\right)\left(N\left(t\right)-1\right)}$$
- *Shannon index* (SH) describes, similar to the Simpson index, the diversity using both the number of different clones as well as the relative abundance of each clone. In information theory it corresponds to the entropy of a discrete memoryless source:
  $$SH\left(t\right)=\;-\sum_{i=1}^{n}r_{i}\text{ln}\left(r_{i}\right)$$
  with $r_{i}=\;\left|N_{i}^{t}\right|/N\left(t\right)$ being the percentage of the corresponding clone relative to the whole population [24, 26].
  *Classical* diversity indices outlined above are instantaneous measures being defined for a single point in time. Motivated by the intention to detect changes in relative clone size, we propose a more sophisticated measure, which uses consecutive data points.
- *Maximum relative clonal expansion* (*mRCE*) is based on the change of the clone abundances considering two (successive) points in time.
  $$\Delta_{i}\left(t\right)=N_{i}^{t}-N_{i}^{t-1}$$

We define a *proportion of newly generated cells s*_*i*_(*t*) by comparing the change in clone size of clone *i* to the sum of this difference for all diminishing, i.e. suppressed, clones:

$$s_{i}\left(t\right)=\frac{\Delta_{i}\left(t\right)}{\sum_{j,\;\Delta_{j}\left(t\right)<0}^{n}\left|\Delta_{j}\left(t\right)\right|}$$

We normalize this measure by the individual clone size $\left|N_{i}^{t}\right|$

$${\bar{s}}_{i}\left(t\right)=\frac{s_{i}\left(t\right)}{\left|N_{i}^{t}\right|}$$

and define the relative expansion of a clone (RCE) as follows

$$RCE_{i}\left(t\right)=\;\{\begin{array}{c} \frac{{\bar{s}}_{i}\left(t\right)}{\sum_{j,\;s_{j}\left(t\right)>0}^{n}{\bar{s}}_{j}\left(t\right)},\;\;if\;s_{i}\left(t\right)>0\\ \frac{{\bar{s}}_{i}\left(t\right)}{\sum_{j,\;s_{j}\left(t\right)<0}^{n}\left|s_{j}\left(t\right)\right|},\;\;else \end{array}$$

Values close to *RCI*_*i*_ = 1 indicate that the expansion of a particular clone *i* was only possible at the expense of *all other* clones, thus indicating a strong clonal dominance. For any given point in time, we therefore characterize a population by the largest value of the $\begin{array}{c} \text{max}\\ \text{n}(RCE_{i}(t)) \end{array}$.

## Results

### Time courses of clonal behavior

**Fig 1:**
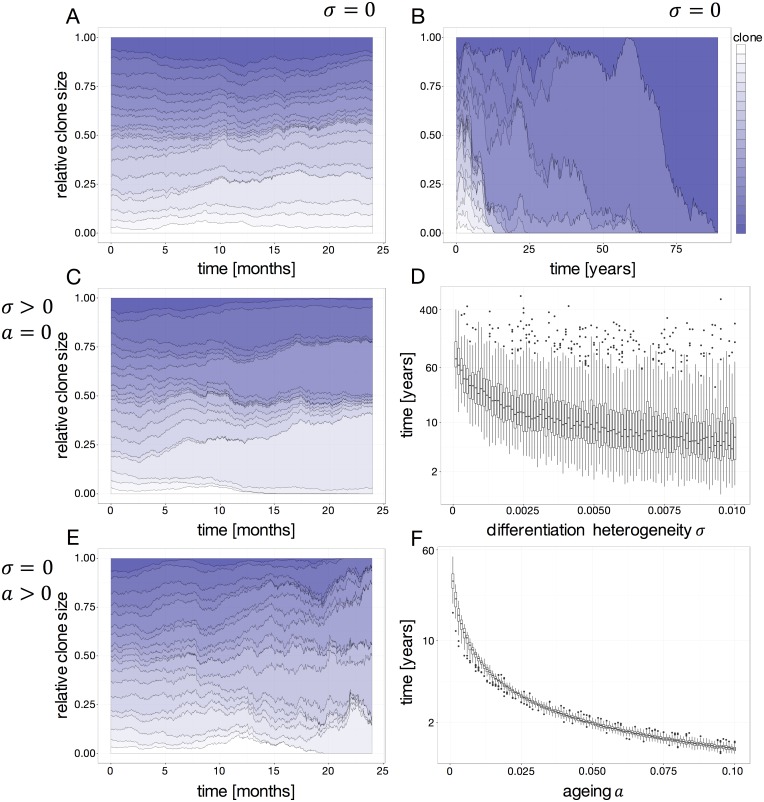
Temporal clonal developments. (A) & (B) Simulation without heterogeneity between the clones (*σ* = 0) for 2 years and till monoclonality is reached (~8 years), respectively. (C) Simulation with no aging effect (*α* = 0) and a simulated heterogeneity of the differentiation rate *d*_*i*_ (*σ* > 0). (D) Average time to reach monoclonality on a logarithmic scale in years vs. the clonal heterogeneity defined by *σ*. (E) Simulation of an aging effect (*α* > 0) and no difference in the differentiation rates *d*_*i*_ between the clones (*σ* = 0). (F) Shows the change in the average time to reach monoclonality depending on the aging effect *α*.

First, we study the clonal behavior within our model under the minimal set of assumptions. Therefore, we initialized the model with a set of identical clones (i.e. no differences in proliferation and differentiation rates). As depicted in Fig 1A, we observe a rather stable coexistence of clones with small fluctuations in the clone size. However, observing the clonal developments beyond a usual mouse’ lifespan, we encounter a well-known phenomenon termed neutral competition and characterized by a slow convergence towards monoclonality (Fig 1B). Even for the case of completely identical clones the stochasticity of division and differentiation events lead to increasing but also decreasing clone sizes. Consequently, smaller clones can vanish over time, finally resulting in a monoclonal situation.

In a second step, we were interested in how inter-clonal variability influences the clonal pattern over time. Therefore, we introduce a heterogeneity in the clone specific differentiation rates *d*_*i*_. As a result we observe only mild changes in the time courses during a normal murine life span (Fig 1C). However, the time to monoclonal conversion (which is usually beyond a normal murine lifespan) decreases for an increasing level of inter-clonal heterogeneity (described by diversity parameter *σ* of the distribution of differentiation rates *d*_*i*_) (Fig 1D).

Third, we investigated the influence of the divisional aging effect defined by the parameter *α*. Heterogeneity in this setting does not only occur between clones (interclonal heterogeneity), but also within clones (intraclonal heterogeneity) due to the differential divisional history of individual cells. The time course in Fig 1E suggests that a high level of interclonal heterogeneity is similar to the above case. As we assume that the cumulative aging effect leads to increased differentiation of the effected cells, it is also plausible that cell clones can become extinct more easily if the differentiation rate decreases the effective rate of self-renewal. The resulting effect on the time to monoclonality is shown in Fig 1F, documenting an even further acceleration.

**Fig 2:**
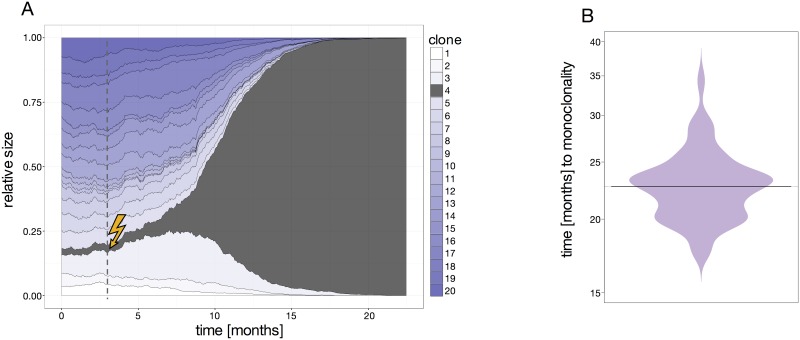
Clonal developments within cancer scenarios. (A) Simulation with one malignant cell (increased proliferation rate *p*) initiated in the third month. The clone in which the mutation occurs is depicted in grey. (B) The average time until clonal dominance beginning at the time point of cancer initiation.

As a fourth case we considered a cancer scenario in which the proliferation rate for one randomly chosen cell is substantial increased. If this cell persists and proliferates in the stem cell compartment, its clonal progeny inherits the same proliferation rate and will finally outcompete all remaining, unaltered cells (Fig 2A).

Although the initialization time of the cancer cell is arbitrary, the proliferative advantage *p*_*m*_ is chosen such that clonal dominance (i.e. 95% of the population derives from the mutated cell type) is achieved on average 23 months after the initiating event. Fig 2B shows a distribution of the time until clonal dominance is reached. The plot documents a substantial variability even for the case that the proliferative advantage is identical for all mutated clones. Similar stochastic fluctuations might occur in leukemia patients, in which the configuration of the healthy cells might also vary and the proliferative advantage of the patient specific leukemic clone will not be identical.

### Quantifying clonality

#### Instantanous measures

**Fig 3:**
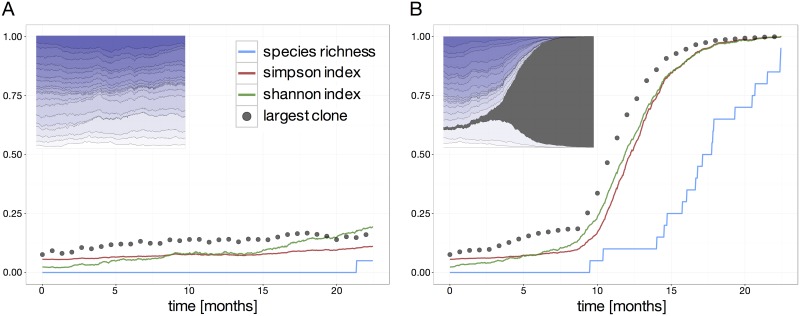
Clonal developments described by classical indices. (A) Time courses of the Simpson and Shannon index as well as species Richness for a non-mutated scenario (clonal pattern in inset). For reference, the size of the largest clone is given by the dotted line. (B) Similar time courses based on a mutation scenario (as provided in the inset). The mutation event occurs after ~3 months.

Diversity measures are designed to quantify differences in abundances at one particular point in time. Typical examples are species richness, Shannon index or Simpson index [23–26]. Time courses of such *classical* measures document changes in these abundances over time. Fig 3 shows a typical time courses for a selection of classical indices measuring the diversity of the clonal contribution based on the examples shown in Figs 1E and 2A, respectively.

Fig 3A depicts a physiological situation which includes intrinsic interclonal heterogeneity and aging. The slight drift in clone sizes (indicated as dotted line, representing the size of the largest clone) is reflected by an increase of both Simpson and Shannon index. Species richness does not show substantial changes as the overall number of detectable clones remains almost unaltered. The picture changes for the pathological situation (Fig 3B). Both Simpson and Shannon index closely follow the increase in size of the malignant clone. The decrease in number of clones is only detected after a certain time delay by a decreasing species richness. However, neither of the measures appear “early responsive” to the sudden increase in size of one particular clone. For these reason, we conclude that these measures are not suited to estimate the risk of clonal dominance or to reliably predict such behaviour.

#### Quantifying changes in the clonal abundances

Clonal dominance is characterized by the increased expansion of a particular clone at the expense of the remaining, non-mutated ones. In order to reliably detect such behavior, we propose a measure that explicitly address clone size differences between consecutive measurements, designated as *relative clonal expansion* (*RCE*). This measure quantifies to which extend the increase in size of one clone is attributed to the total loss of all other clones. *RCE* values close to 1 indicate that one clone expands while all others are suppressed. Given that the pathological clones yield the highest growth rates we only consider the maximum value of this measure over all clones at any given point in time referred as *maximum relative clonal expansion* (*mRCE*). As such, low levels of the *mRCE* measure indicate that multiple clones increase at the expense of multiple competitors, thus representing a physiological competition scenario.

**Fig 4:**
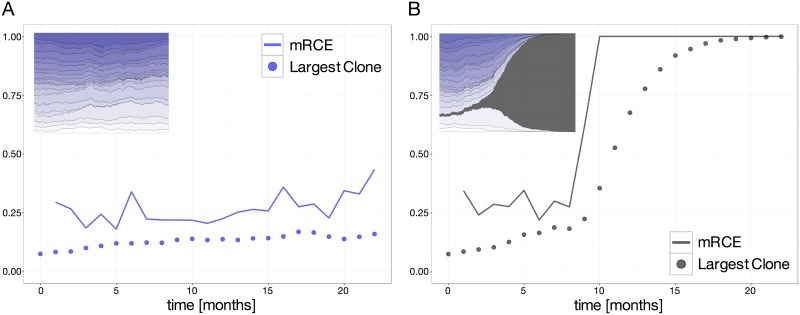
Clonal developments described by *mRCE*. Subfigures show time courses of the *mRCE* (solid line) and the largest clones size (dotted line) for a scenario without (A) and with a mutated clone (B). Both scenarios are initialized with identical initial conditions. In subfigure (B) the mutation is initialized at month 3 after simulation start.

Fig 4 illustrates the responsiveness of the *mRCE* measure for both a physiological and a pathological scenario. Most pronounced, in the pathological case, the *mRCE* measure immediately escapes from the physiological region (*mRCE* ≲ 0.5) as soon as the exponential outgrowth of the malignant clone becomes observable. Note that at this stage none of the suppressed clones are extinct. Considering the potential use as a predictive measure this is a clear advantage compared to the classical measures of clonal diversity that lack this early response.

Sensitivity analysis of the mRCE with respect to the variation of further model parameters showed no distinct influence of the absolute population size and the total number of clones (S1 Fig). It is only the proliferation rate of the malignant cells that shows a strong impact. Intuitively, a more aggressive cancer clone becomes dominant more quickly and can also be detected earlier.

### Predictions

In the previous section we illustrated that the specifically designed *mRCE* measure qualifies as a sensitive predictor for clonal dominance. In the following we evaluate the sensitivity and specificity of the *mRCE* by applying it as a real time measure to approximate future developments and investigate its dependencies on measurement frequency and system intrinsic heterogeneity.

**Fig 5:**
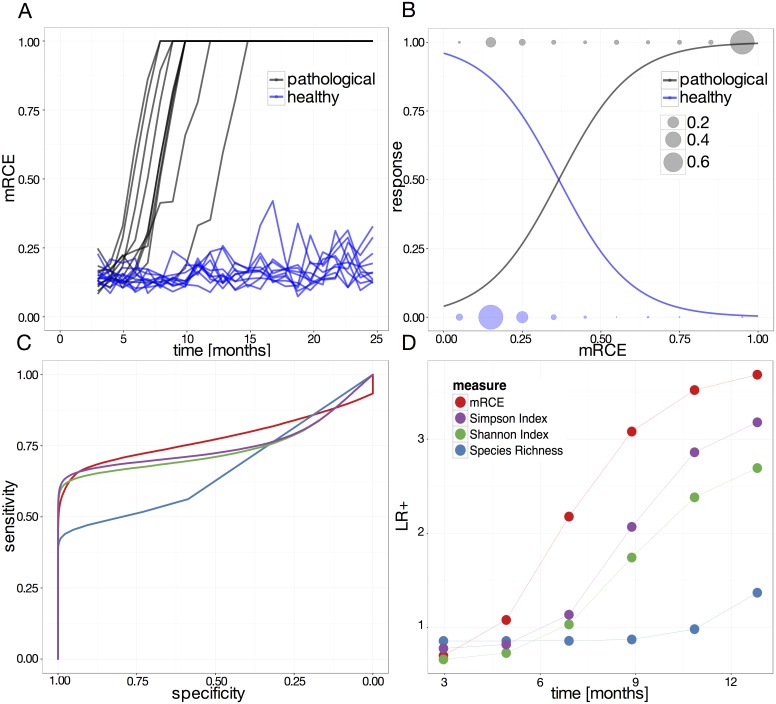
Performance comparison of *mRCE* vs. classical indices. (A) Time courses of *mRCE* values of 10 individual runs of pathological and healthy simulations. (B) The plot shows the distribution of the *mRCE* values for healthy (blue) and pathological (black) contributions (circles). These distributions are used to fit a binomial glm (lines indicate the probability for the occurrence of pathological (black) and physiological (blue) scenarios). (C) AUC comparison of different measures. (D) Positive likelihood ratio comparison over time of different.

Based on 5000 individual runs for healthy and pathological progression scenarios (Fig 5A), we used the observed *mRCE* values at any given point in time to fit a binomial generalized linear model (glm). Cancer initialization times are set to day 0, in order to provide a common starting point. In rare cases, mutated clones are lost early after initialization due to random fluctuations and are excluded from the analysis. Fig 5B shows a representation of the resulting glm, based on the retrospective *mRCE* measure. The circles indicate the abundance of pathological (shown at response = 1) and non-pathological (shown at response = 0) in-silico patients that were identified based on the measured *mRCE* values. For the pathological scenario, *mRCE* values close to 1 are more frequent, whereas most non-pathological *mRCE* values are distributed around 0.2. The glm provides an estimate of the probability that for any measured value of the *mRCE* the corresponding scenario belongs to a pathologic case with fast monoclonal conversion (black curve) or to a physiological scenario without a dominant clone (blue curve). Both probabilities are equal for *mRCE* ≈ 0.33 (point of intersection, Fig 5B), while the probability for facing a pathological case is already 4 times higher for a *mRCE ≈* 0.5 compared to the physiological scenario. Typically, levels of *mRCE* at 0.5 are already reached even if the dominant, potentially malignant clone takes up only about 12% of the total cell population. These findings strongly support the high sensitivity of the introduced *mRCE* measure and mark it as a classification predictor for fast monoclonal conversion.

Fig 5C shows the receiver operating characteristic (ROC) curve and the corresponding area under the curve (AUC) values of the different measures. Clearly the *mRCE* measure outperforms the established ones in terms of specificity as well as sensitivity. Whereas Shannon and Simpson indices perform almost equal, species richness is considerably worse in terms of the AUC. Since species richness is considering only the extinction of clones it is insensitive for the detection of rapid expansion and thus lead to a prediction time delay (compare Fig 3B).

Fig 5D illustrates the positive likelihood ratio (defined as *sensitivity*/(1 − *specificity*) of the outlined methods for predicting the existence of a leukemic clone as a function of the time after leukemia initiation. Consistently, all measures correctly indicate increasing evidence for leukemia manifestation. However, the *mRCE* measure is clearly superior to the instantaneous measures as values above LR+ = 1 (thereby associating the test result with disease) are detected much earlier.

We further investigated, whether the performance of the classical diversity measures can be increased by taking into account changes between consecutive measurement, similar to the approach for the mRCE. Although this “retrospective view” increases the prediction accuracy, the mRCE still performs better than the classical measures (S2 Fig). This has to be accounted to the design of the mRCE index, which describes changes in the size of *individual* clones while the classical measures only describe the population as a whole.

### Influences of different sources of heterogeneity

**Fig 6:**
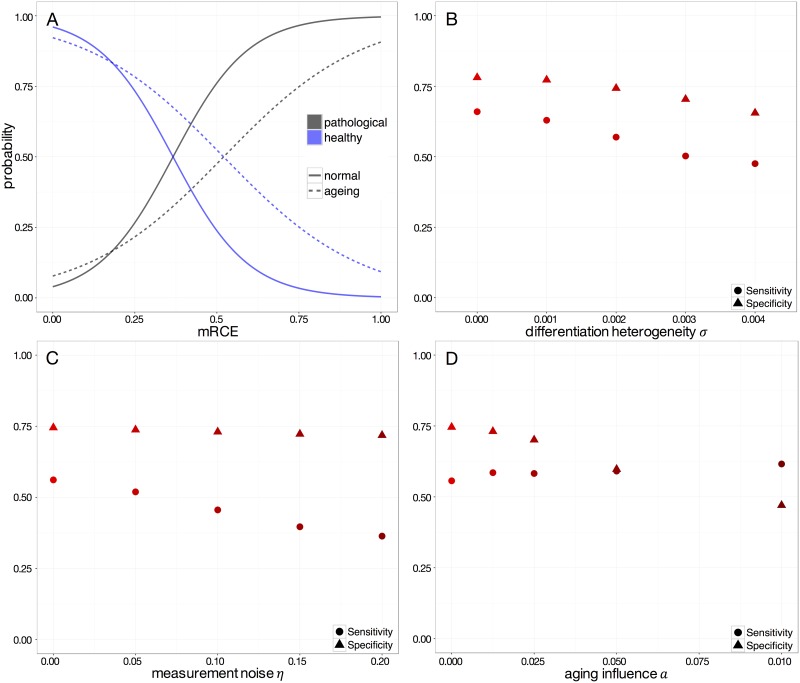
Influence of different sources of heterogeneity and uncertainty on the performance of the *mRCE* measure. (A) The binomial glms of a homogenous (solid line, *σ* = 0) and a non-homogenous model (dashed line, *σ* = 0.03) (B–D) Sensitivity and Specificity at *t* = 7 months after cancer initialization for different values of the differentiation heterogeneity *σ* (B), measurement noise *η* (C) and aging influence *α* (D).

So far we only investigated an idealized scenario of initially almost equally sized clones without any measurement errors. We would expect that the prediction quality decreases for more diverse, real-world settings. To investigate those settings, we first studied the case of an increased interclonal heterogeneity by allowing for larger differences in the clonal differentiation rate, described by the variance measure *σ*. Fig 6A compares this setting to the idealized case. Fitting the glm to the new, heterogeneous training data one observes that the threshold of the *mRCE* level that allows for a consolidated prediction of the clonal dominance scenario is shifted. While for the previous scenario the probability for correctly predicating a pathological versus a physiological scenario was 4 times increased for *mRCE* ≈ 0.5, this level is now reached only at about *mRCE* ≈ 0.8. Similarly, Fig 6B documents the decline in the sensitivity and specificity of the *mRCE* prediction with increasing interclonal heterogeneity. In fact, the further we increase this heterogeneity the closer the system approaches the leukemic case in which one dominating clone outcompetes all others. As the scenarios become less distinguishable, sensitivity and specificity monotonically decline.

In the next scenario we study, how measurement errors could influence the prediction accuracy. For simplicity, we assume a white noise of magnitude *η* overlying all clone size measurements. Accessing sensitivity and specificity of the *mRCE* measure for a given time point after cancer initiation in Fig 6C, one observes a moderate decline of these quality measures. In fact, the sensitivity almost stays constant for the considered noise levels *η*.

We also investigated to which extend the measurement accuracy depends on the size of a randomly sampled subset of all cells. Unsurprisingly, we observe a decrease of the LR+ (Subfigure A in S3 Fig) as the limited accessibility of the clonal composition makes it more difficult to reliable detect changes in the clone size. However, this effect is compensated if, for a constant number of labeled clones, the overall number of cells (either directly in the compartment or even down-stream) is increased (Subfigure B in S3 Fig).

We observe a similar loss of prediction accuracy for the scenario in which we introduce an even more pronounced aging effect *α*. Here we assume that the individual proliferative history of each cell determines its tendency for differentiation and ultimately the exit from the stem cell compartment. Although the specificity of the *mRCE* measure declines for a stronger aging effect, this is not the case for the sensitivity. For all the outlines scenarios it should be pointed out that sensitivity and specificity of the model predictions are not independent. In fact, each new model scenario requires the fitting of an individual glm on which the predictions will be based. Therefore, the decline of either the sensitivity or the specificity indicate a decrease of the overall performance.

Our model simulations demonstrate that increased levels of clonal heterogeneity or measurement errors lead to a loss of prediction accuracy and a less clear separation of the healthy and pathological situation. Thereby, identification of early tumor growth is limited, although the proposed *mRCE* measure shows a remarkable robustness.

## Discussion

Describing the dynamics of individual clones in hematopoiesis is a prerequisite to understand the underlying mechanisms of cellular competition and the process during leukemic transformation. We used a simple agent-based model to describe a self-renewing stem cell pool and a corresponding pool of differentiated cells. Similar to clonal tracking experiments (e.g. using cellular barcodes or integration sites), we are able to label HSCs with a unique marker which is inherited to its daughter cells in order to establish an identifiable clone.

**Fig 7:**
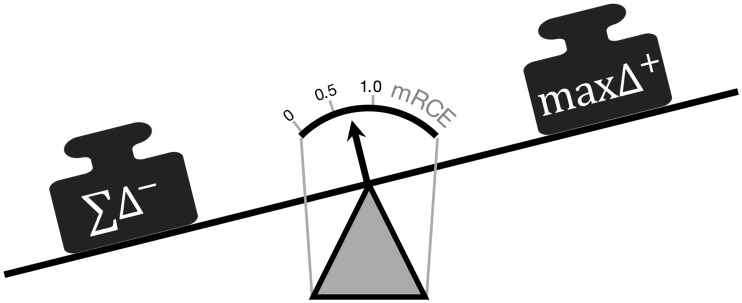
Scale representation of *mRCE* measurement. *ΣΔ*^−^ denotes the sum of all shrinking clones and *maxΔ*^+^ the clone with the highest expansion between two consecutive points in time. Only the case of one dominantly expanding clone balances the scale and indicates a high risk for rapid monoclonal conversion (*mRCE* = 1).

We also applied different already established diversity measures, such as species richness or Simpson index that are adapted from ecology [23–25], to quantify temporal clonal behavior. Since these measures are defined for one particular point in time only, they are not able to systematically detect *changes* in clonal abundances over time. As a consequence, we suggest a measure, referred to as *maximum Relative Clonal Expansion* (*mRCE*), which is sensitive to temporal changes. The principal idea is to evaluate changes in the relative clone sizes between consecutive time points. The *mRCE* quantifies the proportion of the shrinking clones, denoted as Σ*Δ*^−^, compared to the increase of the clone with the largest net growth (max*Δ*^+^). Fig 7 illustrates the relationship between the two influential factors of the *mRCE* are depicted. For the extreme case that *all* shrinking clones are suppressed due to the expansion of *one* single clone, the *mRCE* value approaches 1. This scenario is frequently seen if one clone possesses a substantial growth advantage, such as a cancer clone. In the contrary, for competition between equally potent cell clones, the *mRCE* takes values ≪1. This sensitivity for the detection of one primarily expanding clone makes the suggested measure ideally suited to prospectively distinguish physiological from pathological behaviors. In fact, the information we gain by considering more than just one point in time, lead to the necessary sensitivity to prospectively detect rapid clonal outgrowth at very early stages which is the key for a beneficial prediction of malignant cases. We also showed that the predictive power remains even for more realistic scenarios including interclonal heterogeneity and measurement noise.

Within the simplified model setup, cancer outgrowth is achieved by initializing a substantial growth advantage within one mutated cell and thereby seeding this characteristic for all derived progeny. However, in a real world scenario, acquisition of a mutational growth advantage may result from a sequential accumulation of various different mutations. In any case, pathologically relevant situations occur once a functionally impaired clone takes over the entire system. If this clone is trackable, the *mRCE* is a promising measure to detect this altered competition. In contrast, the *mRCE* is not suited to identify the functional impairment of a potentially dominating, healthy clone. Thereby, our model points to a dilemma in the early detection of hematological tumors. Especially in older individuals, neutral or mild clonal competition inevitably results in a clonal conversion process. However, this conversion towards monoclonality is not necessarily linked to a pathological transformation but may result from a physiological condition. In this respect, the clonal composition itself is not a reliable marker of cancer. Instead, we argue that the “speed” of clonal conversion is a better indicator of the fitness advantage that commonly goes along with cancer outgrowth although the functional impairment cannot be proven on the clonal level.

Transferring our suggested strategy into a clinical protocol the questions remains how to correctly chose appropriate time intervals for the successive measurement. In general, larger intervals will detect larger differences in clone size, thereby leading to more accurate predictions. In contrast, for short time intervals tumor growth might be too marginal to be detected. This results in a dilemma as an accurate detection should be available as early as possible. We advocate the view that the velocity of the expected leukemic growth should be the reference to schedule the measurements: while acute leukemias will rapidly chance the clonal repertoire, shorter time intervals are warranted, while for chronic and slowly expanding tumors, longer time intervals are sufficient.

Ongoing efforts to closely monitor gene therapy patients [19] and recent achievements in the robust and quantitative identification of viral integration sides [27] allow for clonal tracking in patients with a high risk of leukemic transformation within the marked cell population [28, 29]. Our suggested *mRCE* measure is uniquely suited to operate in this setting and points towards the occurrence of clonal conversion process at a very early stage, prior to observing other clinical markers such as altered blood counts. Our model based approach is a first step to demonstrate the suitability of such measures in controlled setting and thereby provides the basis for application to clinical data that should become available in the near future.

## Supporting Information

S1 Fig Influence of model parameter for prediction quality of mRCE. We show LR+ value (defined as sensitivity/(1 − specificity)) of the mRCE as a measure of the prediction accuracy for changes of different model parameters. We varied the population size (1000 cells = low, red / 4000cells = high, light red), the number of clones (10 = low, green / 40 = high, light green) and the cancer proliferation rate ((9.9 days)^-1^ slow, blue/ (3.3 days)^-1^ = high, light blue). While the population size and the clone number rarely influence the quality of the read-out, the proliferation rate of the malignant cells has a strong impact. Intuitively, a more aggressive cancer clone becomes dominant more quickly, and is also earlier detectable.
(TIF)

S2 Fig Retrospectivity improves the classical measures. Retrospectivity can be applied to classical measures by considering the change of two consecutive time points. Clearly, mRCE still is superior in terms of LR+ compared to the classical measures. However, compared to Fig 5 of the main text, the prediction accuracy is increased especially at earlier time intervals (< 9 months).
(TIF)

S3 Fig Prediction accuracy of the mRCE for different, randomly sampled subsets. Predictions based on different samples sizes (lines). Dots describe the relative abundance of the largest clones averaged over all cancer time courses. Percentages refer to the sizeof a randomly chosen subset of the original polyclonal population (A) For a system with K = 2000 cells, the decrease of the size of the sampled subsets limits the earlier detectability of the leukemic growth. (B) For an increase in the total number of cells (K = 20000) the adverse effect of the sampling procedure is compensated.
(TIF)

S1 Code Pseudocode of the aging based modelling process. First all cells have to be initialized according to their clonal properties (proliferation rate, differentiation rate, replicative age). Afterwards we compute an update step (1 day) for all cells using the explicit Euler method. First, for every cell it is decided whether it proliferates according to a maximal proliferation rate of the clone and the actual number of cells. Second, for every cell it is decided whether it differentiates within this time step according to a differentiation rate, which dependents on the clonal differentiation rate and the number of prior cell divisions. In case a cell is proliferating, the cell is duplicated and all properties are transferred to the new sibling. In case a cell is differentiating it is deleted from the proliferating compartment. After updating all cells, the process starts over for the next time step until the time reaches the configured maximum time.
(PDF)
