# Supplementary figures and images for: Model Based Analysis of Clonal Developments Allows for Early Detection of Monoclonal Conversion and Leukemia
Source: PLoS One. 2016 Oct 20;11(10):e0165129. doi: 10.1371/journal.pone.0165129 (PMC5072636; doi:10.1371/journal.pone.0165129)

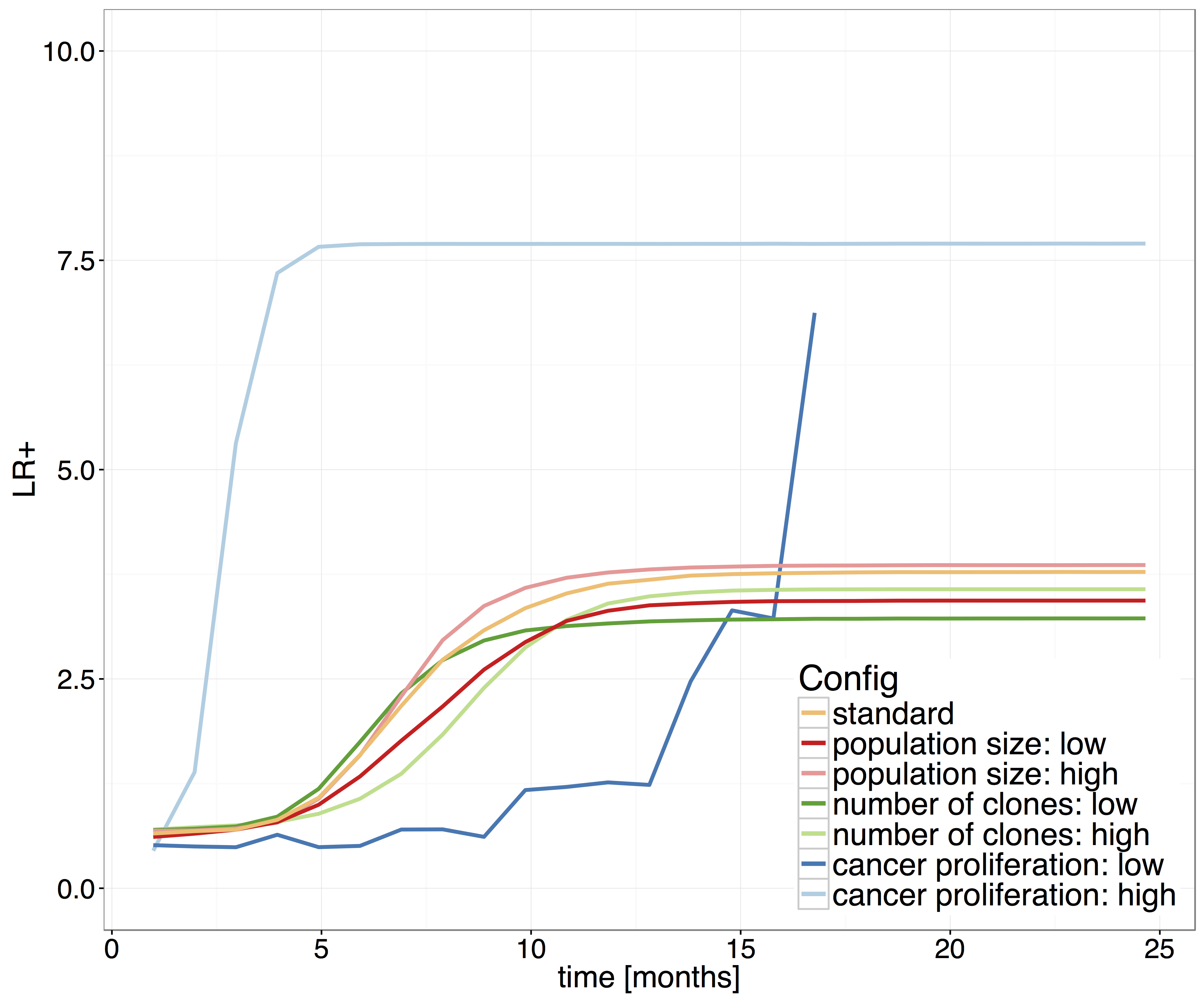

Supplement: S1 Fig — We show LR+ value (defined as sensitivity/(1 − specificity)) of the mRCE as a measure of the prediction accuracy for changes of different model parameters. We varied the population size (1000 cells = low, red / 4000cells = high, light red), the number of clones (10 = low, green / 40 = high, light green) and the cancer proliferation rate ((9.9 days)-1 slow, blue/ (3.3 days)-1 = high, light blue). While the population size and the clone number rarely influence the quality of the read-out, the proliferation rate of the malignant cells has a strong impact. Intuitively, a more aggressive cancer clone becomes dominant more quickly, and is also earlier detectable. (TIF) [file pone.0165129.s001.tif]

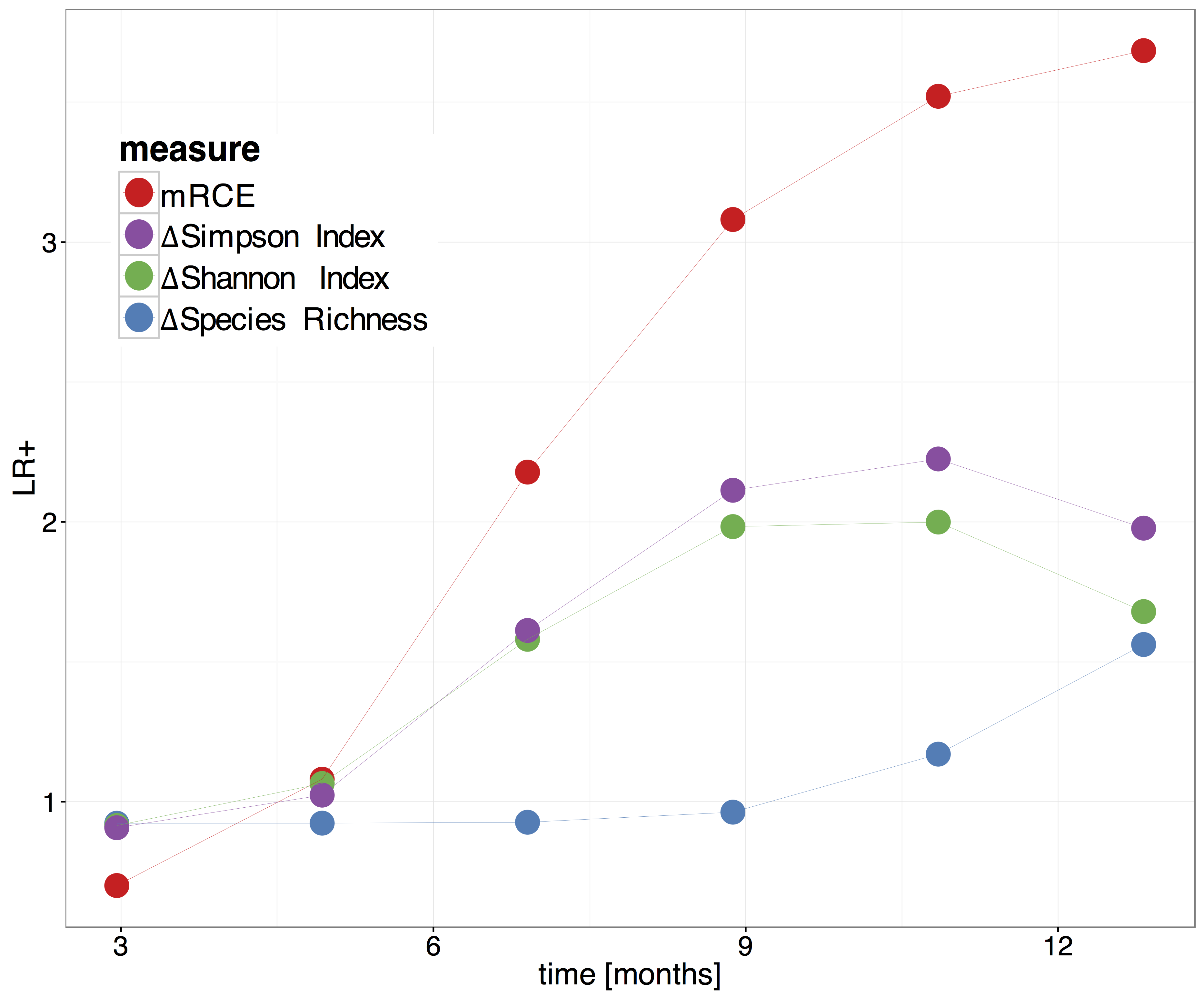

Supplement: S2 Fig — Retrospectivity can be applied to classical measures by considering the change of two consecutive time points. Clearly, mRCE still is superior in terms of LR+ compared to the classical measures. However, compared to Fig 5 of the main text, the prediction accuracy is increased especially at earlier time intervals (< 9 months). (TIF) [file pone.0165129.s002.tif]

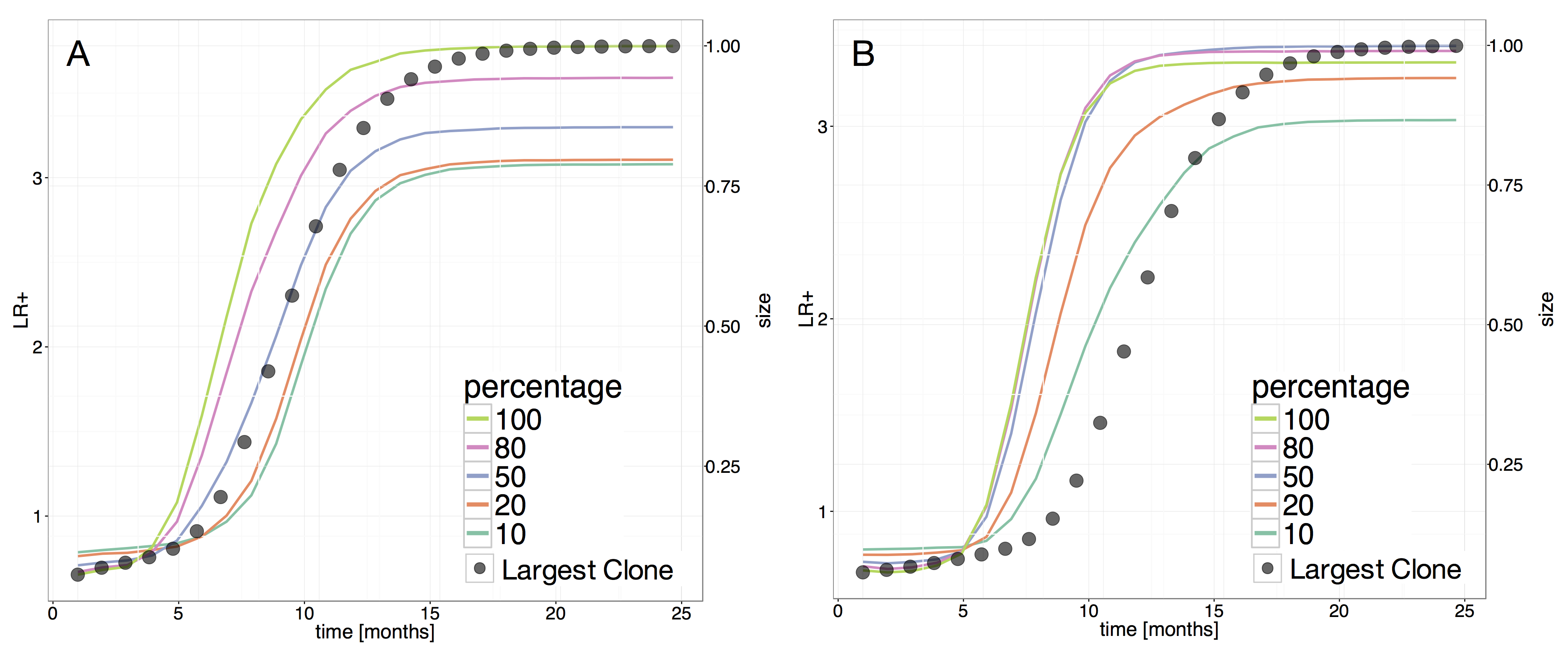

Supplement: S3 Fig — Predictions based on different samples sizes (lines). Dots describe the relative abundance of the largest clones averaged over all cancer time courses. Percentages refer to the sizeof a randomly chosen subset of the original polyclonal population (A) For a system with K = 2000 cells, the decrease of the size of the sampled subsets limits the earlier detectability of the leukemic growth. (B) For an increase in the total number of cells (K = 20000) the adverse effect of the sampling procedure is compensated. (TIF) [file pone.0165129.s003.tif]
