# Supplementary material for: Model Based Analysis of Clonal Developments Allows for Early Detection of Monoclonal Conversion and Leukemia
Source: PLoS One. 2016 Oct 20;11(10):e0165129. doi: 10.1371/journal.pone.0165129 (PMC5072636; doi:10.1371/journal.pone.0165129)
Supplement: S1 Code — First all cells have to be initialized according to their clonal properties (proliferation rate, differentiation rate, replicative age). Afterwards we compute an update step (1 day) for all cells using the explicit Euler method. First, for every cell it is decided whether it proliferates according to a maximal proliferation rate of the clone and the actual number of cells. Second, for every cell it is decided whether it differentiates within this time step according to a differentiation rate, which dependents on the clonal differentiation rate and the number of prior cell divisions. In case a cell is proliferating, the cell is duplicated and all properties are transferred to the new sibling. In case a cell is differentiating it is deleted from the proliferating compartment. After updating all cells, the process starts over for the next time step until the time reaches the configured maximum time. (PDF) [file pone.0165129.s004.pdf]

```
initialize max_days
initialize cells according to properties of clones
for days = 1 up to max_days
  for each cell
    if cell proliferate?
       duplicate cell and inherit clonal identity
        increase cell division counter by 1
    if cell(s) differentiate?
        delete cell(s) from proliferating compartment
```
